# Prevalence and incidence of hypertension in a heavily treatment-experienced cohort of people living with HIV in Uganda

**DOI:** 10.1371/journal.pone.0282001

**Published:** 2023-02-17

**Authors:** Dathan M. Byonanebye, Mark N. Polizzotto, Rosalind Parkes-Ratanshi, Joseph Musaazi, Kathy Petoumenos, Barbara Castelnuovo

**Affiliations:** 1 Kirby Institute, University of New South Wales, Sydney, Australia; 2 School of Public Health, Makerere University, Kampala, Uganda; 3 The Australian National University, Canberra, Australia; 4 Infectious Diseases Institute, Kampala, Uganda; 5 Cambridge Institute of Public Health, University of Cambridge, Cambridge, United Kingdom; Azienda Ospedaliera Universitaria di Perugia, ITALY

## Abstract

**Introduction:**

The effect of long-term exposure to antiretroviral therapy (ART) on hypertension in sub-Saharan Africa remains unclear. We aimed to determine the prevalence and incidence of hypertension in people living with HIV (PLWH) with more than 10 years of ART in Uganda.

**Methods:**

The analysis was performed within a cohort of adult PLWH with more than 10 years of ART at an HIV clinic in Kampala, Uganda. Participants were eligible for this analysis if they had ≥2 follow-up visits. Hypertension was defined as two consecutive systolic blood pressure (SBP) measures greater than 140 mmHg and/or diastolic blood pressure (DBP) greater than 90 mmHg, and/or documented diagnosis and/or the initiation of antihypertensives. We determined the proportion of PLWH with hypertension at baseline and used multivariable logistic regression to determine the factors associated with prevalent hypertension. To determine the incidence of hypertension, follow-up began from the cohort baseline date and was censored at the last clinic visit or date of the event, whichever occurred earlier. Multivariable Poisson regression was used to determine the adjusted incidence rate ratios (aIRR) of hypertension according to demographic, ART, and clinical characteristics.

**Results:**

Of the 1000 ALT participants, 970 (97%) had ≥2 follow-up visits, and 237 (24.4%) had hypertension at baseline. The odds of prevalent hypertension were 1.18 for every 5-year increase in age (adjusted odds ratio (aOR) 1.18, 95% CI 1.10–1.34) and were higher among males (aOR 1.70, 95% CI 1.20–2.34), participants with diabetes mellitus (aOR 2.37, 95% CI 1.10–4.01), obesity (aOR 1.99, 95% CI 1.08–3.60), high cholesterol (aOR 1.47, 95% CI 1.16–2.01), and those with prior exposure to stavudine (aOR 2.10, 95% CI 1.35–3.52), or nevirapine (aOR 1.90, 95% CI 1.25–3.01). Of the 733 participants without hypertension at baseline, 116 (15.83%) developed hypertension during 4671.3 person-years of follow-up (incidence rate 24.8 per 1000 person-years; 95% CI 20.7–29.8). The factors associated with incident hypertension were obesity (adjusted incidence rate ratio (aIRR) 1.80, 95% CI 1.40–2.81), older age (aIRR 1.12 per 5-year increase in age, 95% CI 1.10,1.25), and renal insufficiency (aIRR1.80, 95% CI 1.40–2.81).

**Conclusion:**

The prevalence and incidence of hypertension were high in this heavily treated PLWH cohort. Therefore, with increasing ART coverage, HIV programs in SSA should strengthen the screening for hypertension in heavily treated PLWH.

## Introduction

The successful scale-up of antiretroviral therapy (ART) has improved the overall survival of people living with HIV (PLWH) globally and in sub-Saharan Africa (SSA) [1], although survival still lags that of people without HIV [2]. This improvement in survival has contributed to an epidemiological transition in the cause of mortality in PLWH away from AIDS-related opportunistic infections towards non-communicable diseases, including cardiovascular diseases [3, 4]. Globally, the risk of cardiovascular disease is twice as high in PLWH than in people without HIV. In some SSA countries, more than 15% of the cardiovascular burden occurs in PLWH [5]. However, it is unclear whether the increasing cardiovascular burden in PLWH is attributable to ageing and/or the increasing prevalence of cardiovascular disease risk factors, including hypertension. Hypertension is an important independent risk factor for cardiovascular disease, accounting for up to 44% of cardiovascular events in some HIV cohorts [15]. The cardiovascular risk attributable to hypertension is higher in black people than in other ethnicities [6]; however, there is little data on hypertension in heavily treated PLWH in SSA.

Despite increasing ART coverage among PLWH in SSA [7], the impact of long-term ART exposure on cardiovascular risk factors, such as hypertension, has not been fully described [8]. The relationship between ART exposure and hypertension was initially investigated in the Multicenter AIDS Cohort, in which it was demonstrated that exposure to ART for more than two years was associated with a higher risk of hypertension [9]. While this analysis was conducted before ART became widely available, subsequent studies in the modern ART era have also demonstrated a greater risk of hypertension in ART-experienced versus ART-naïve PLWH. A recent systematic review reported higher systolic and diastolic blood pressures of 4.52 mmHg and 3.17 mmHg, respectively, in ART-experienced PLWH than in treatment-naive individuals [10]. The effect of ART on blood pressure appears to be more detrimental in sub-Saharan Africa; a recent study showed that ART for at least three months is associated with an increase in systolic and diastolic blood pressure of 7.85 mm Hg and 7.45 mm Hg, respectively [11]. The prevalence of hypertension among PLWH increased by 10% between 2007 and 2017 [12], possibly due to increasing ART coverage and overall survival.

It is unclear whether the increase in blood pressure following ART initiation is sustained with prolonged exposure to ART and whether the exposure translates to higher rates of hypertension. Additionally, prior analyses have linked older antiretroviral drugs such as nevirapine, indinavir/ritonavir, and stavudine to a higher risk of hypertension [13, 14]. Until recently, these agents were the cornerstones of ART in SSA [15, 16] although it is unclear whether the risk posed by these agents persists in PLWH who switch to contemporary ART regimens, the regimens currently recommended for HIV treatment. Therefore, in this analysis, we sought to determine the prevalence and incidence of hypertension and its relationship with exposure to specific antiretroviral therapy in a Ugandan cohort of PLWH with long durations of ART.

## Methods

The analysis was conducted within the antiretroviral treatment long-term (ALT) cohort (ClinicalTrials.gov #: NCT02514707), which has previously been described [17, 18]. Briefly, the ALT study is an ongoing single-centre prospective cohort of 1000 PLWH on ART for at least 10 years at baseline. Cohort participants were recruited from the Infectious Diseases Institute (IDI) HIV clinic in Kampala, Uganda, between 2014 and 2015. Because of prolonged exposure to ART at baseline (>10 years), the participants in this cohort were considered heavily treatment-experienced since HIV resistance testing is limited in resource-limited settings. At the cohort baseline, data on HIV treatment, and other covariates, including blood pressure measurements and hypertension treatment, were collected from an existing clinic health information management system. Participants in the cohorts are evaluated annually, blood pressure measurements are taken, and treatment for hypertension and other co-comorbidities, including hypertension, is documented. Blood pressure measurement is standardised at all visits, and readings are taken by qualified nurses using calibrated blood pressure machines, consistent with international guidelines [19].

Data on complications associated with HIV infection and ART is also collected. Laboratory tests (HIV RNA viral load, CD4 count, and creatinine level) are also performed at every visit, whereas lipid tests were only available at baseline. HIV treatment for participants is standardised and follows the World Health Organization (WHO) and Uganda ART guidelines [20, 21]. All PLWH receive a three-drug ART regimen consisting of two nucleos(t)ide reverse transcriptase inhibitors (NRTIs) and non-nucleoside reverse transcriptase inhibitors (NNRTI) as the first-line treatment or protease inhibitors (PIs) as the second-line treatment. In 2018, dolutegravir (DTG) was introduced in Uganda as a part of a three-drug regimen for all patients receiving ART. Patients already on ART were recommended to switch from PIs or NNRTIs to DTG-containing regimens, and the process of switching all participants is underway.

### Participant selection and inclusion criteria into analysis datasets

We included all participants in the cohort with at least two blood pressure measurements recorded at two different follow-up visits. Participants with fewer than two follow-up visits were excluded because hypertension diagnosis requires at least two measurements taken at two different visits [19]. In addition, individuals with hypertension at baseline were excluded from the hypertension incidence analysis. The baseline date for all analyses was the date of enrolment in the cohort (i.e., cohort baseline).

### Definitions and analysis endpoints

Hypertension was defined as systolic blood pressure (SBP) ≥ 140 mmHg and/or diastolic blood pressure (DBP) ≥ 90 mmHg on two consecutive visits or initiation of antihypertensives including angiotensin-converting enzyme inhibitors (ACEIs). This definition has been used in other analyses of HIV cohorts in SSA [22–24] and a prior analysis of the ALT cohort [18]. The definition is also consistent with international Hypertension diagnosis and management guidelines, which recommend two consecutive abnormal blood pressure measurements [19]. Therefore, prevalent hypertension, determined at baseline, was defined as two consecutive systolic blood pressure (SBP) measures greater than 140 mmHg and/or diastolic blood pressure (DBP) greater than 90 mmHg, and/or documented diagnosis and/or the initiation of antihypertensives within one year before and up to one month after the baseline date. The other covariates were also defined as consistent with a prior analysis of this cohort [18]. Diabetes mellitus was defined as a clinical diagnosis and/or random blood glucose levels ≥11.1 mmol/L based on point-of-care glucose testing and/or the initiation of antidiabetic medication, consistent with other analyses in HIV cohorts [25, 26]. Hepatitis B infection was defined as a positive hepatitis B surface antigen test result, which is the screening strategy recommended by WHO for high-burden countries [27]. The Chronic Kidney Disease Epidemiology Collaboration (CKD-EPI) equation was used to determine the estimated glomerular filtration rate (eGFR), and renal insufficiency was defined as eGFR <90 mL/min/1.73 m^2^ [28].

### Statistical analyses

We determined the proportion of PLWH with hypertension at baseline and compared the characteristics of PLWH with hypertension versus those without hypertension. Descriptive statistics for ordinal and categorical variables were computed as frequencies and percentages, and continuous variables were summarised as medians (interquartile ranges, [IQR]). Multivariable logistic regression was used to determine the factors associated with hypertension at baseline. We then performed univariable analysis followed by multivariable Poisson regression to determine hypertension incidence rate ratios (aIRR) and their corresponding 95% confidence intervals (CI) according to exposure to demographic, HIV-infected, and clinical characteristics. While fitting the Poisson regression, the logarithm of person-years of follow-up was included in the model as an offset term to account for the observation time of individuals.

The overall incidence of hypertension was determined and stratified according to demographic, metabolic, and HIV-related factors. Follow-up began from the cohort baseline date and was right censored at the last available study visit or the date of the event, whichever occurred earlier. The factors that were considered in the multivariable model include sex, age, smoking and alcohol status, calendar year, prior AIDS, total cholesterol (TCHOL), triglycerides (TRIG), low-density lipid cholesterol (LDL), body mass index (BMI), diabetes, HIV RNA and CD4 counts, and eGFR and prior exposure to individual antiretroviral drugs (before baseline). These factors were selected a priori based on published data linking them to hypertension [14, 22, 24, 29, 30]. All variables, except age, were modelled as categorical variables, and the categories were selected so that clinically meaningful conclusions could be made. For example, the BMI categories followed the WHO categories [31], and blood pressure categories are based on the European Association for cardiology categorisation for blood pressure [32]. The CD4 categories are also based on the Centers for Disease Control and Prevention (CDC) categories for immunosuppression status [33] while lipids were categorised following the thresholds for cardiovascular risk [34]. All variables in this analysis were fixed at baseline, and the regression models were manually fitted. In the backward selection process, variables with a p-value <0.25 at the univariable stage were considered in the multivariable analysis. All variables dropped from the multivariate model were assessed to determine if they were confounders (i.e., causes >10% change in effects when added in the model), in which case they were retained in the adjusted model. The goodness-of-fit test was used to determine the fitness of the model with the confounder versus the one without the confounder and the one with the smallest Akaike information criterion were considered the better fitting. Specifically, BMI and age have consistently been shown to be confounders for hypertension [14, 22, 24, 29, 30]; their confounding potential was checked, and the variables were consistently included in the model. Variables, other than confounders, that were considered for multivariable analysis but dropped during backward elimination due to non-significance (p<0.05) were later added one at a time to determine adjusted estimates for these variables. We checked for multicollinearity in the variables included in the final model using a variance inflation factor (VIF) cut-off of 5.0. Odds ratios (95% confidence intervals (CI)) and incidence rate ratio (95% CI) were used to determine the association between variables and prevalent and incident hypertension, respectively, and a two-sided p-value <0.05 was considered statistically significant. The final logistic and Poisson regression models were evaluated to ensure compliance with the respective model assumptions. The Wald chi-square test was used to determine whether the final model was statistically significant.

Where data were missing for a variable, an unknown category was assigned, and the variable was fitted as a categorical variable. To determine the impact of missing data and the robustness of estimates, we used the regular Little’s test [35], to determine if missing data were missing completely at random (MCAR) and if multiple imputation was necessary. Data for this analysis were prepared using SAS Enterprise Guide software version 8.3 release 5 (SAS Institute Inc., Cary, NC, USA) and analysed with Stata version 16.0 (StataCorp, College Station, Texas, USA). Participants in the ALT cohort provided written informed consent, and the study received ethical approval from the Joint Clinical Research Council Research Ethics Committee (JCRC REC) and Uganda National Council of Science and Technology (UNCST; Approval #: UNCST Folio Number: HS 1586 24th 03 2014).

## Results

### Cohort description and characteristics of study participants

**Fig 1:**
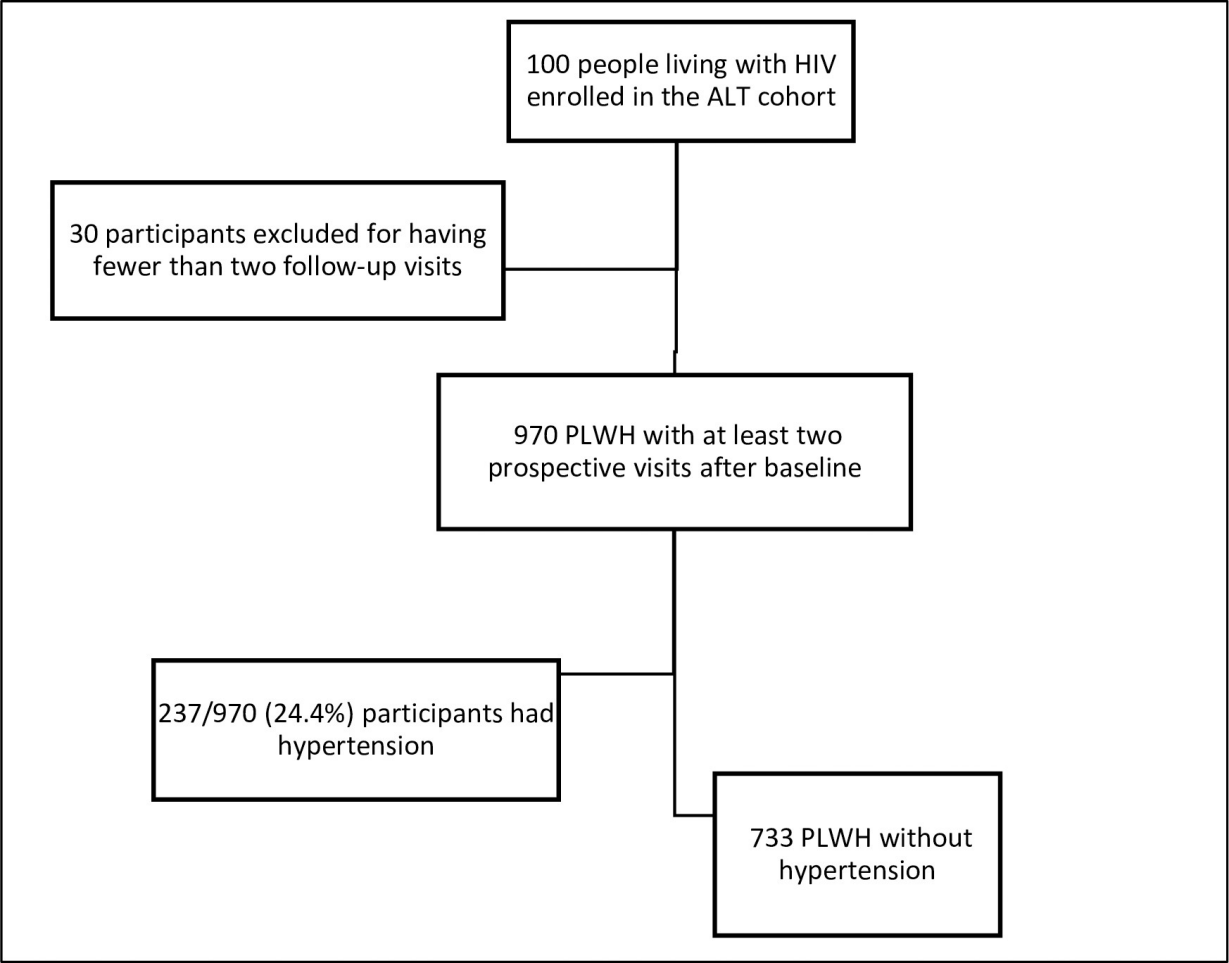
Participant inclusion for hypertension and weight gain analysis.

A total of 1000 participants were enrolled in the ALT cohort, 970 of whom had at least two follow-up visits (with at least two blood pressure measurements) and were included in the analysis to determine the prevalence of hypertension (Fig 1). The median number (interquartile range, IQR) of follow-up visits was 5 (4–6), and all visits were annual. The 30 participants who were excluded because they had fewer than two follow-up visits had comparable ages (median age: 45.0; IQR 40.0,51.0) but were mainly male (53%).

### Prevalence of hypertension

Of the 970 participants included in the prevalence analysis, 237 (24.4%) had hypertension at cohort enrolment (baseline). Overall, the median age was 45.4 (IQR 40.4–50.5) years and there were more females (61.8%) than males. A total of 466 participants (48.0%) had experienced an AIDS event before enrolment in the cohort; 960/970 (99.0%) had HIV RNA levels <200 copies/mL, and the median CD4 cell count was 509 (IQR,365–684). The baseline smoking rate was 2.2%. The characteristics of participants with and without hypertension at enrolment are presented in Table 1.

**Table 1:** Characteristics of participants with versus without hypertension at cohort enrolment (n = 970).

Variable		Prevalent Hypertension		No Hypertension*		All	
		N	%	N	%	n	%
		237	24.4	733	75.57	970	100
Sex	Male	101	42.6	270	36.8	371	38.3
	Female	136	57.4	463	63.2	599	61.8
Prior AIDS	Yes	113	47.7	353	48.2	466	48.0
	No	124	52.3	380	51.8	504	52.0
Hepatitis B infection	Positive	8	3.4	31	4.2	39	4.0
	Negative	229	96.6	702	95.8	931	96.0
Smoking Status	Current	3	1.3	18	2.5	21	2.2
	Prior	50	21.1	149	20.3	199	20.5
	Never	184	77.6	566	77.2	750	77.3
Alcohol Status	Current	52	21.9	188	25.7	240	24.7
	Prior	184	77.6	530	72.3	714	73.6
	Never	1	0.4	15	2.1	16	1.7
Diabetes mellitus	Yes	21	8.9	14	1.9	35	3.6
	No	216	91.1	719	98.1	935	96.4
NRTI backbone	TDF/3TC	67	28.3	243	33.2	310	32.0
	AZT/3TC	164	69.2	486	66.3	650	67.0
	ABC/3TC	4	1.7	4	0.6	8	0.8
	Other	2	0.84	0	0	2	0.2
ART class	NNRTIs	198	83.5	582	79.4	780	80.4
	PIs	39	16.5	151	20.6	190	19.6
**Variable **		**Median (IQR)**	**n missing (%)**	**Median (IQR)**	**n missing (%)**	**Median (IQR)**	**n missing (%)**
Age (years)		47.4 (42.4,52.7)	0 (0)	44.7 (40.3,50.3)	0 (0)	45.4 (40.4,50.5)	0 (0)
Systolic Blood Pressure		130 (120,146)	0 (0)	120 (110,125)	0 (0)	120 (110,130)	0 (0)
Diastolic Blood Pressure		80 (70,90)	0 (0)	70 (69,80)	0 (0)	70 (70,80)	0 (0)
Body mass index (Kg/M^2^)		23.1 (20.3,26.0)	5 (2.11)	22.1 (19.7,25.0)	8 (1.1)	22.4 (19.8,25.3)	13 (1.3)
eGFR (mL/min/1.73 m2)		116 (103,128)	86 (36.3)	122.9 (109,130)	325 (44.3)	121.4 (107,130)	411 (42.4)
HIV RNA (copies/mL)		19 (19,19)	0 (0)	19 (19,19)	0 (0)	19 (19,19)	0 (0)
Baseline CD4 (cells/μL)		518 (378,689)	0 (0)	508 (359,683)	0 (0)	509 (365,684)	0 (0)
Nadir CD4 (cells/μL)		391 (275,507)	0 (0)	401 (290,528)	0 (0)	399 (289,523)	0 (0)
TCHOL (mmol/L)		4.9 (4.3,5.7)	2 (0.8)	4.7 (4.0,5.3)	12 (1.6)	4.7 (4.1,5.4)	14 (1.4)
LDL (mmol/L)		2.8 (2.2,3.3)	2 (0.8)	2.6 (2,3.2)	11 (1.5)	2.6 (2.0,3.2)	13 (1.3)
HDL (mmol/L)		1.2 (1.0,1.5)	2 (0.8)	1.2 (1.0,1.5)	11 (1.5)	1.2 (0.98,1.5)	13 (1.3)
TRIG (mmol/L)		1.4 (1.1,1.9)	94 (39.7)	1.3 (0.9,1.8)	358 (48.8)	1.3 (1.0,1.8)	452 (46.6)
Number of Follow-up visits		5 (4,6)	0 (0)	5 (4,6)	0 (0)	5 (4,6)	0 (0)
Baseline date (mm/yy)		02/15 (08/14,05/15)	0 (0)	08/14 (05/15,06/15)	0 (0)	03/15 (08/14,06/15)	0 (0)
**Exposure to ART class/NRTIS (years)**		**Median (IQR)**	**n exposed (%)**	**Median (IQR)**	**n exposed (%)**	**Median (IQR)**	**n exposed (%)**
Cumulative exposure to NNRTIs		9.4 (7.7,9.6)	234 (98.7)	9.3 (4.9,9.6)	721 (98.4)	9.3 (5.4,9.6)	955 (98.5)
Cumulative exposure to PIs		6.3 (3.0,7.3)	37 (15.6)	4.9 (2.2,6.7)	139 (19.0)	5.4 (2.4,6.9)	176 (18.1)
Cumulative exposure to ddI		5.9 (2.7,7.1)	11 (4.6)	6.8 (5.2,7.4)	28 (3.8)	6.0 (5.1,7.4)	39 (4.0)
Cumulative exposure to d4T		2.7 (2.1,3.2)	201 (84.8)	2.6 (2.0,3.1)	570 (77.8)	2.6 (2.1,3.1)	771 (79.5)
Cumulative exposure to AZT		6.5 (4.9,7.0)	210 (88.6)	6.4 (2.4,7.0)	684 (93.3)	6.5 (3.0,7.0)	894 (92.2)
Cumulative exposure to TDF		5.1 (2.4,6.9)	64 (27.0)	3.5 (1.7,6.1)	230 (31.4)	3.6 (1.8,6.3)	294 (30.3)
Cumulative exposure to EFV		9.3 (6.2,9.6)	204 (86.1)	9.2 (4.6,9.6)	592 (80.8)	9.3 (4.9,9.6)	796 (82.1)
Cumulative exposure to NVP		2.2 (0.1,7.6)	69 (29.1)	1.6 (0.1,8.4)	278 (37.9)	1.6 (0.1,8.2)	347 (35.8)
Cumulative exposure to LPV		6.4 (5.3,7.1)	33 (13.9)	6.0 (4.0,7.2)	102 (13.9)	6.1 (4.1,7.2)	135 (13.9)
Cumulative exposure to ATV		2.1 (1.6,2.4)	8 (3.4)	1.4 (0.4,2.1)	42 (5.7)	1.6 (0.5,2.2)	50 (5.2)

Note: *733 participants without hypertension at baseline were included in the incidence analysis. n means the number of participants; AIDS-prior AIDS-defining event; NNRTIs-non nucleoside reverse transcriptase inhibitors; NRTIs-nucleos(t)ide reverse transcriptase inhibitors, ART-antiretroviral therapy, eGFR-estimated glomerular filtration rate, PI-protease inhibitors, TDF- tenofovir disoproxil fumarate; EFV-efavirenz, LPV-lopinavir; ATV-atazanavir; ddI-didanosine; d4t-stavudine; AZT-zidovudine; NVP-nevirapine; LPV-lopinavir; RTV-ritonavir; ABC-abacavir; 3TC-lamivudine; TRIG-triglycerides; HDL-high-density lipoprotein; LDL-low-density lipoprotein cholesterol; TCHOL-total cholesterol; mm/yy means month followed by the year of the baseline date.

Compared with those without hypertension, participants with hypertension at cohort enrolment were older, more likely to be male, had diabetes mellitus, had higher total cholesterol levels, and had more prolonged exposure to stavudine and nevirapine. After adjustment for confounders, the odds of prevalent hypertension were higher among males (aOR 1.70, 95% CI 1.20–2.34), as well as in participants with diabetes mellitus (2.37, 95% CI 1.10–4.01), obesity (aOR 1.99, 95% CI 1.08–3.60), higher total cholesterol (aOR 1.47, 95% CI 1.16–2.01), and prior exposure to stavudine (aOR 2.10, 95% CI 1.35–3.52), or nevirapine (aOR 1.90, 95% CI 1.25–3.01). In addition, the odds of prevalent hypertension were higher in participants with missing triglyceride levels. Finally, the odds of hypertension were 1.18 (CI 1.10–4.01) for every 5-year increment in the cohort enrolment age (Table 2).

**Table 2:** Factors associated with prevalent hypertension in the ALT cohort at baseline (n = 970).

Variable	Variable Categories	Crude OR (95% CI)	p-value	Adjusted OR (95% CI)	p-value
**Sex**	**Female**	**Ref**		**Ref**	
	**Male**	**1.27 (0.95,1.72)**	**0.112**	**1.70 (1.20,2.34)**	**0.002**
*Alcohol History*	*Never*	*Ref*			
	*Current*	*4*.*15 (0*.*54*,*32*.*14)*	*0*.*173*	*1*.*10 (0*.*19*,*12*.*04)*	*0*.*856*
	*Prior*	*5*.*21 (0*.*68*,*39*.*7)*	*0*.*111*	*1*.*67 (0*.*20*,*14*.*42)*	*0*.*698*
**Diabetes mellitus**	**No DM**	**Ref**		**Ref**	
	**DM**	**2.97 (1.48,5.95)**	**0.002**	**2.37 (1.10,4.01)**	**0.011**
**Age, Per 5 years increment**		**1.20 (1.10,1.31)**	**0.000**	**1.18 (1.10,1.34)**	**0.003**
**BMI (Kg/M2)**	**<18.5**	**0.98 (0.62,1.54)**	**0.922**	**0.91 (0.56,1.50)**	**0.567**
	**18.5–24.9**	**Ref**		**Ref**	
	**25–29.9**	**1.33 (0.92,1.92)**	**0.130**	**1.47 (0.98,2.30)**	**0.075**
	**>29.9**	**1.66 (1.15,2.87)**	**0.047**	**1.99 (1.08,3.60)**	**0.031**
*GFR (mL/min/1*.*73 m2)*	*<90*	*1*.*60 (0*.*85*,*2*.*99)*	*0*.*142*	*1*.*13 (0*.*61*,*2*.*46)*	*0*.*765*
	*≥90*	Ref		*Ref*	
	*Missing*	*0*.*75 (0*.*55*,*1*.*02)*	*0*.*063*	*1*.*43 (0*.*95*,*2*.*09)*	*0*.*078*
HIV RNA (copies/mL)	*<200*	*1*.*74 (0*.*81*,*3*.*76)*	*0*.*159*	*1*.*78 (0*.*71*,*4*.*00)*	*0*.*173*
	*≥200*	Ref		Ref	
*Baseline TRIG (mmol/L)*	*<1*.*7*	*Ref*		*Ref*	
	*1*.*7–2*.*2*	*1*.*07 (0*.*58*,*1*.*95)*	*0*.*837*	*0*.*83 (0*.*50*,*1*.*60)*	*0*.*680*
	*≥2*.*3*	*1*.*41 (0*.*86*,*2*.*30)*	*0*.*169*	*1*.*17 (0*.*69*,*1*.*96)*	*0*.*574*
	*Missing*	*0*.*74 (0*.*53*,*1*.*03)*	*0*.*071*	*2*.*94 (2*.*00*,*4*.*69)*	*<0*.*001*
*Baseline LDL (mmol/L)*	*<2*.*6*	*Ref*		*Ref*	
	*2*.*6–3*.*3*	*1*.*11 (0*.*79*,*1*.*56)*	*0*.*547*	*0*.*98 (0*.*65*,*1*.*54)*	*0*.*861*
	*≥3*.*4*	*1*.*50 (1*.*02*,*2*.*19)*	*0*.*037*	*1*.*10 (0*.*49*,*1*.*99)*	*0*.*900*
	*Missing*	*0*.*63 (0*.*14*,*2*.*88)*	*0*.*549*	*0*.*60 (0*.*19*,*4*.*72)*	*0*.*660*
**Baseline TCHOL (mmol/L)**	**<5.2**	**Ref**			
	**≥5.2**	**1.57 (1.15,2.12)**	**0.004**	**1.47 (1.16,2.01)**	**0.008**
	**Missing**	**0.60 (0.13,2.71)**	**0.505**	**0.48 (0.46,2.80)**	**0.460**
**Prior exposure to d4T**	**Never exposed**	**Ref**		**Ref**	
	**Prior Exposure**	**1.60 (1.08,2.37)**	**0.020**	**2.10 (1.35,3.52)**	**<0.001**
*Prior exposure to TDF*	*Never exposed*	*Ref*		*Ref*	
	*Prior Exposure*	*0*.*81 (0*.*58*,*1*.*12)*	*0*.*200*	*0*.*76 (0*.*61*,*1*.*34)*	*0*.*489*
*Prior exposure to EFV*	*Never exposed*	*Ref*		*Ref*	
	*Prior Exposure*	*0*.*73 (0*.*51*,*1*.*07)*	*0*.*104*	*0*.*75 (0*.*51*,*1*.*13)*	*0*.*144*
**Prior exposure to NVP**	**Never exposed**	**Ref**		**Ref**	
	**Prior Exposure**	**1.47 (0.98,2.21)**	**0.062**	**1.90 (1.25,3.01)**	**0.002**
*Prior exposure to ATV*	*Never exposed*	*Ref*		*Ref*	
	*Prior Exposure*	*0*.*61 (0*.*28*,*1*.*31)*	*0*.*203*	*0*.*86 (0*.*40*,*2*.*14)*	*0*.*710*

Note: OR-odds ratio, AIDS-prior AIDS-defining event; BMI-body mass index; eGFR-estimated glomerular filtration rate; TDF-tenofovir disoproxil fumarate; EFV-efavirenz; ATV-atazanavir; d4T-stavudine; NVP-nevirapine, TRIG-triglycerides, HDL-high-density lipoprotein; LDL-low-density lipoprotein cholesterol; TCHOL-total cholesterol. Covariates prior AIDS, hepatitis B infection status, smoking history, prior exposure to lopinavir (LPV), abacavir (ABC), didanosine (ddI), high-density lipoprotein, baseline year, and baseline and nadir CD4 counts were not significant and were not confounders in the univariable analysis (P>0.2) and were not considered in the multivariable analysis. The significant variables in the final multivariable model are in bold, whereas the insignificant variables are in italics.

### Incidence of hypertension

After excluding 237 participants with hypertension at enrolment, 733 participants were included in the incidence analysis. Of the 733 included participants, the baseline ART regimen consisted of NNRTIs in 583 (79.5%), and the remaining 150 (20.5%) received PI-based regimens. The NRTI backbones were zidovudine/lamivudine (AZT/3TC) (486, 66.3%), tenofovir disoproxil fumarate (TDF) with emtricitabine or lamivudine (TDF/XTC) (243, 33.2%), and abacavir/lamivudine (ABC/3TC) (4, 0.6%) (Table 3).

**Table 3:** Characteristics of participants without hypertension at baseline (n = 733).

		Incident Hypertension		No incident Hypertension		All	
		n	%	n	%	n	%
		116	15.8	617	84.2	733	100
Sex	Male	43	37.1	227	36.8	270	36.8
	Female	73	62.9	390	63.2	463	63.2
Prior AIDS	Yes	46	39.7	307	49.8	353	48.2
	No	70	60.3	310	50.2	380	51.8
Hepatitis B infection	Positive	4	3.5	27	4.4	31	4.2
	Negative	112	96.6	590	95.6	702	95.8
Smoking Status	Current	3	2.6	15	2.4	18	2.5
	Prior	16	13.8	133	21.6	149	20.3
	Never	97	83.6	469	76.0	566	77.2
Alcohol Status	Current	24	20.7	164	26.6	188	25.7
	Prior	89	76.7	441	71.5	530	72.3
	Never	3	2.6	12	1.9	15	2.1
Diabetes mellitus	Yes	3	2.6	11	1.8	14	1.9
	No	69	59.5	358	58.0	427	58.3
	Missing	44	37.9	248	40.2	292	39.8
NRTI backbone	TDF/3TC	30	25.9	213	34.5	243	33.2
	AZT/3TC	86	74.14	400	64.8	486	66.3
	ABC/3TC	0	0	4	0.7	4	0.6
ART regimen	NNRTIs	103	88.8	480	77.8	583	79.5
	PIs	13	11.2	137	22.2	150	20.5
** **		**Median (IQR)**	**n missing (%)**	**Median (IQR)**	**n missing (%)**	**Median (IQR)**	**n missing (%)**
Age (years)		45.6 (42.2,51.7)	0 (0)	44.4 (40,49.7)	0 (0)	44.7 (40.3,50.3)	0 (0)
Systolic Blood Pressure		130 (120,140)	0 (0)	112 (110,120)	0 (0)	120 (110,125)	0 (0)
Diastolic Blood Pressure		80 (70,85.5)	0 (0)	70 (67,74)	0 (0)	70 (69,80)	0 (0)
BMI (Kg/M^2^)		23.2 (21.1,27.1)	0 (0)	21.8 (19.5,24.9)	8 (1.30)	22.1 (19.7,25.0)	8 (1.1)
GFR (mL/min/1.73 m2)		121 (103,126)	43 (37.1)	123 (110,130)	282 (45.7)	122.9 (109,130)	325 (44.3)
HIV RNA (copies/mL)		19 (19,19)	0 (0)	19 (19,19)	0 (0)	19 (19,19)	0 (0)
Baseline CD4 (cells/μL)		534 (373.5,679)	0 (0)	506 (350,687)	0 (0)	508 (359,683)	0 (0)
Nadir CD4 (cells/μL)		411 (290,581.5)	0 (0)	399 (290,519)	0 (0)	401 (290,528)	0 (0)
TCHOL (mmol/L)		4.7 (4.2,5.4)	3 (2.6)	4.6 (4.0,5.3)	9 (1.5)	4.7 (4.0,5.3)	12 (1.6)
LDL (mmol/L)		2.7 (2.1,3.2)	3 (2.6)	2.6 (2.0,3.1)	8 (1.3)	2.6 (2.0,3.2)	11 (1.5)
HDL (mmol/L)		1.3 (1.0,1.5)	3 (2.6)	1.2 (1.0,1.5)	8 (1.3)	1.2 (1.0,1.5)	11 (1.5)
TRIG (mmol/L)		1.5 (1.0,2.2)	49 (42.2)	1.3 (0.9,1.7)	309 (50.1)	1.3 (0.9,1.8)	358 (48.8)
Number of Follow-up visits		5 (5,6)	0 (0)	5 (4,6)	0 (0)	5 (4,6)	0 (0)
Baseline date (mm/yy)		02/15 (08/14,06/15)	0 (0)	03/15 (08/14,06/15)	0 (0)	08/14 (05/15,06/15)	0 (0)
**Exposure to ART class (years)**		**Median (IQR)**	**n exposed (%)**	**Median (IQR)**	**n exposed (%)**	**Median (IQR)**	**n exposed (%)**
Cumulative exposure to NNRTIs		9.4 (9.1,9.6)	115 (99.1)	9.3 (3.9,9.6)	606 (98.2)	9.3 (4.9,9.6)	721 (98.4)
Cumulative exposure to PIs		7.9 (3.0,8.1)	11 (9.5)	4.6 (2.2,6.5)	128 (20.8)	4.9 (2.2,6.7)	139 (19.0)
Cumulative exposure to ddI		7.1 (6.0,7.6)	6 (5.2)	6.0 (5.2,7.4)	22 (3.6)	6.2 (5.2,7.4)	28 (3.8)
Cumulative exposure to d4T		2.6 (2.1,3.1)	93 (80.2)	2.6 (2.0,3.0)	477 (77.3)	2.6 (2.0,3.1)	570 (77.8)
Cumulative exposure to AZT		6.7 (4.1,7.0)	109 (94.0)	6.3 (2.0,7.0)	575 (93.2)	6.4 (2.4,7.0)	684 (93.3)
Cumulative exposure to TDF		3.1 (1.1,6.9)	25 (21.6)	3.5 (1.8,6.0)	205 (33.2)	3.5 (1.7,6.1)	230 (31.4)
Cumulative exposure to EFV		9.4 (9.1,9.6)	97 (83.6)	9.2 (3.8,9.5)	495 (80.2)	9.2 (4.6,9.6)	592 (80.8)
Cumulative exposure to NVP		0.7 (0.1,7.6)	33 (28.5)	1.6 (0.1,8.4)	245 (39.7)	1.6 (0.1,8.4)	278 (37.9)
Cumulative exposure to LPV		8.1 (7.8,8.4)	9 (7.8)	5.8 (3.9,6.8)	93 (15.1)	6.0 (4.0,7.2)	102 (13.9)
Cumulative exposure to RTV		7.9 (3.0,8.4)	11 (9.5)	4.6 (2.2,6.5)	128 (20.8)	4.9 (2.2,6.7)	139 (19.0)
Cumulative exposure to ATV		0.8 (0.4,1.3)	2 (1.7)	1.5 (0.4,2.2)	40 (6.5)	1.4 (0.4,2.1)	42 (5.7)

Note: AIDS means prior AIDS-defining event, NRTI-nucleos(t)ide reverse transcriptase inhibitors, ART-antiretroviral therapy, BMI-body mass index, eGFR-estimated glomerular filtration rate, NNRTIs-non-nucleoside reverse transcriptase inhibitors, PI-protease inhibitors, TDF-tenofovir disoproxil fumarate, EFV-efavirenz, LPV-lopinavir, ATV-atazanavir, ddI-didanosine, d4T-stavudine, AZT-zidovudine, NVP-nevirapine, LPV-lopinavir, RTV-ritonavir, ABC-abacavir, 3TC-lamivudine, TRIG-triglycerides, HDL-high-density lipoprotein, LDL-low-density lipoprotein cholesterol, TCHOL-total cholesterol, mm/yy-month/year.

A total of 116 participants (15.8%) developed hypertension during 4671.3 person-years of follow-up, with an incidence rate of 24.8 per 1000 person-years (95% CI 20.7–29.8). The risk of developing hypertension increased with increasing follow-up (S1 Fig). Of the 116 participants with incident hypertension, 93 (80.2%) initiated antihypertensive therapy. Participants with obesity were 62% more likely to develop hypertension than those with normal body mass index (BMI) (aIRR 1.80, 95% CI 1.40–2.81), while the risk of hypertension was 1.80 higher in participants with eGFR <90 mL/min/1.73 m^2^ compared to those with normal renal function (aIRR 1.89, 95% CI 1.20–4.56). The risk of hypertension also increased by 12% (95% CI, 10%–25%) for each 5-year increase in age. There was no evidence to suggest that the association between BMI and hypertension differed according to sex (interaction P = 0.565) or age (interaction P = 0.496). In addition, participants with diastolic blood pressure >80 mmHg and/or systolic blood pressure >120 mmHg were more likely to develop hypertension during follow-up. There was no relationship between exposure to individual antiretroviral agents and the incidence of hypertension (Table 4).

**Table 4:** Factors associated with of incident hypertension in the ALT cohort (n = 733).

Variable	Variable categories	(Events/PYFU)	Incidence Rate/1000 PY (95% CI)	Crude IRR (95% CI)	p-value	Adjusted IRR (95% CI)	p-value
Prior AIDS	Yes	46/2263	20.3 (15.2,27.1)	0.70 (0.48,1.01)	0.060	0.93 (0.58,1.20)	0.579
	No	70/2408.3	29.1 (23.0,36.7)	Ref		Ref	
NRTI	TDF/3TC	30/1538.5	19.5 (13.6,27.9)	Ref		Ref	
	AZT/3TC	86/3103.6	27.7 (22.4,34.2)	1.42 (0.94,2.15)	0.098	1.24 (0.83,1.90)	0.340
	ABC/3TC	0/29.2	0	-		-	
ART Class	NNRTIs	103/3705.6	27.8 (22.7,33.4)	2.04 (1.14,3.63)	0.016	1.50(0.90,2.70)	0.150
	**PIs**	**13/962.6**	**13.5 (7.8,23.3)**	** Ref**	** **	** Ref**	** **
**Age**	**Per 5 years**	**116/4671.3**	**24.8 (20.7,29.8)**	**1.19 (1.07,1.32)**	**0.002**	**1.12 (1.10,1.25)**	**0.009**
**BMI (Kg/m2)**	**<18.5**	**12/638.5**	**18.8 (10.7,33.1)**	**0.82 (0.44,1.51)**	**0.516**	**0.89(0.50,1.64)**	**0.670**
	**18.5–24.9**	**66/2864.3**	**23.0 (18.1,29.3)**	** Ref**	** **	** Ref**	** **
	**25–29.9**	**22/888.4**	**24.8 (16.3,37.6)**	**1.07 (0.66,1.74)**	**0.770**	**0.87 (0.53,1.41)**	**0.567**
	**>29.9**	**16/280**	**57.1 (35.0,93.3)**	**2.48 (1.44,4.28)**	**0.001**	**1.80 (1.40,2.81)**	**0.009**
**GFR (mL/min/1.73 m2)**	**<90**	**8/193.1**	**41.4 (20.7,82.8)**	**1.56 (0.75,3.25)**	**0.236**	**1.89 (1.20,4.56)**	**0.017**
	**≥90**	**65/2446.8**	**26.6 (20.8,33.9)**	**Ref**	** **	** Ref**	** **
	**Missing**	**43/2031.4**	**21.2 (15.7,28.5)**	**0.80 (0.54,1.17)**	**0.248**	**0.70 (0.49,1.10)**	**0.112**
Nadir CD4 (cells/μL)	<200	9/411.6	21.9 (11.4,42.0)	0.69 (0.34,1.41)	0.310	0.66 (0.36,1.38)	0.257
	200–349	33/1379	23.9 (17.0,33.7)	0.76 (0.48,1.18)	0.221	0.74 (0.48,1.17)	0.170
	350–500	29/1460.2	19.9 (13.8,28.6)	0.63 (0.39,1.00)	0.050	0.80(0.51,1.26)	0.250
	>500	45/1420.5	31.7 (23.7,42.4)	Ref		Ref	
Baseline Year	2014	52/1829.8	28.4 (21.7,37.3)	1.26 (0.88,1.82)	0.213	1.19 (0.80,1.77)	0.277
	2015	64/2841.5	22.5 (17.6,28.8)	Ref		Ref	
**Systolic blood pressure (mmHg)**	**<120**	**17/2399.5**	**7.1 (4.4,11.4)**	**Ref**	** **	** Ref**	** **
	**120–129**	**27/1289.1**	**20.9 (14.4,30.5)**	**2.96 (1.61,5.42)**	**<0.001**	**2.34 (1.31,4.33)**	**0.006**
	**≥130**	**72/982.7**	**73.3 (58.2,92.3)**	**10.34 (6.1,17.54)**	**<0.001**	**5.43(3.20,9.41)**	**<0.001**
**Diastolic blood pressure (mmHg)**	**<80**	**43/3512.4**	**12.2 (9.1,16.5)**	**Ref**	** **	** Ref**	** **
	**80–84**	**42/705.6**	**59.5 (44,80.5)**	**4.86 (3.18,7.44)**	**<0.001**	**2.83 (1.82,4.44)**	**<0.001**
	**≥85**	**31/453.3**	**68.4 (48.1,97.2)**	**5.59 (3.52,8.86)**	**<0.001**	**2.39 (1.43,4.10)**	**<0.001**
Prior exposure to PIs	Never exposed	105/3787.7	27.7 (22.9,33.6)	Ref		Ref	
	Prior Exposure	11/883.6	12.4 (6.9,22.5)	0.45 (0.24,0.84)	0.012	0.79 (0.43,1.56)	0.256
Prior exposure to TDF	Never exposed	91/3227.9	28.2 (23.0,34.6)	Ref		Ref	
	Prior Exposure	25/1443.4	17.3 (11.7,25.6)	0.61 (0.39,0.96)	0.031	0.69 (0.56,1.38	0.200
Prior exposure to EFV	Never exposed	98/3596.8	27.2 (22.4,33.2)	Ref		Ref	
	Prior Exposure	18/1074.5	16.8 (10.6,26.6)	0.61 (0.37,1.02)	0.058	0.98 (0.57,1.58)	0.771
Prior exposure to LPV	Never exposed	107/4020	26.6 (22.0,32.2)	Ref		Ref	
	Prior Exposure	9/651.3	13.8 (7.2,26.6)	0.52 (0.26,1.03)	0.059	0.59(0.34,1.36)	0.276
Prior exposure to ATV	Never exposed	114/4415.8	25.8 (21.5,31.0)	Ref		Ref	
	Prior Exposure	2/255.5	7.8 (2.0,31.3)	0.30 (0.07,1.23)	0.094	1.00(0.70, 1.41)	0.936

Note: * PYFU-person-years of follow-up; AIDS-prior AIDS-defining event; NRTI-nucleos(t)ide reverse transcriptase inhibitors; ART-antiretroviral therapy; BMI-body mass index; GFR-glomerular filtration rate; PI-protease inhibitors; TDF-tenofovir disoproxil fumarate; EFV-efavirenz; LPV-lopinavir; ATV-atazanavir; ABC-abacavir; 3TC-lamivudine. "Prior exposure to PIs" was collinear with "Prior exposure to LPV" (VIF = 6.9). However, these variables were insignificant in the final model (even when included one at a time), and they were dropped. The adjusted estimates for these variables were obtained by adding the variables back into the adjusted model, one at a time. Covariates sex, prior AIDS, hepatitis B infection status, smoking history, alcohol history, diabetes mellitus, prior exposure to didanosine (ddI), stavudine (d4t), zidovudine (AZT), nevirapine (NVP), non-nucleoside reverse transcriptase inhibitors (NNRTIs), baseline lipid values (triglycerides, high-density lipoprotein, low-density lipoprotein cholesterol and total cholesterol), HIV RNA, and baseline CD4 counts were all non-significant and were not confounders in the univariable analysis (P>0.2) and were not considered in the multivariable analysis. The significant covariates are indicated in bold.

### Data completeness and sensitivity analysis

Overall, there was high data completeness on most variables at baseline except eGFR (411/970, 42.4%) and triglycerides (452/970, 46.6%). The regular Little’s MCAR test gives a χ2 distance of 42.86 with 32 degrees of freedom, P = 0.095. The null hypothesis for Little’s MCAR test is that the data are missing completely at random (MCAR). Therefore, we do not reject the null hypothesis, and the test provides evidence that the missing data in the six variables of interest are not MCAR under significance level 0.05. Since data were missing completely at random, there was no justification for using imputing missing data.

## Discussion

This prospective cohort study is among the few to determine the prevalence and incidence of hypertension and its association with exposure to antiretrovirals in PLWH with long ART exposure in SSA. The prevalence of hypertension at enrolment was 24.4% and the factors associated with hypertension were older age, male sex, diabetes mellitus, obesity, and elevated total cholesterol levels. Compared with PLWH without hypertension at enrolment, the odds of prevalent hypertension were also higher in individuals with prior exposure to stavudine and nevirapine. The overall cumulative incidence was 15.8%, with an incidence rate of 24.8 per 1000 person-year of follow-up. Incident hypertension was associated with older age, renal insufficiency, obesity, and blood pressure levels higher than optimal, but not with exposure to antiretroviral drugs.

The incidence of hypertension in the present analysis is higher than that in a previous analysis in the same clinic, which reported a prevalence of 15.1% and an incidence of 19 cases per 1000 person-years eight years before our analysis [36]. However, participants in the present analysis are approximately 10 years older and with longer ART exposure. Increasing rates of hypertension in PLWH have been similarly reported in another cohort of people living with HIV in Uganda [37]. Together, these results suggest increasing rates of hypertension in PLWH in SSA, probably due to ageing and increases in other hypertension risk factors such as ART exposure. While direct comparisons between cohorts cannot be made, the presented incidence of hypertension is within the range of 19 and 54 cases per 1000 person-years, as reported by two studies in Uganda and South Africa, respectively [23, 36]. However, other cohorts in SSA have reported higher incidence rates than those in our analysis [22, 24]. The difference in hypertension rates between cohorts may reflect differences in cohorts as well as differences in hypertension screening. Therefore, HIV programs in SSA should integrate hypertension screening and management into existing ART delivery innovations, as integrated models of hypertension/HIV care have been demonstrated to be feasible even in public facilities in SSA [38, 39].

Our findings are also consistent with analyses of other cohorts in SSA that reported male sex, old age, low nadir CD4 count, and obesity as risk factors for hypertension [22]. Therefore, PLWH with these characteristics should be closely monitored. Previous exposure to nevirapine and stavudine has also been associated with risk or progression in several studies [13, 22, 40, 41]. Although contemporary ART regimens are increasingly available in most SSA countries, participants with prior exposure to these toxic agents should be targeted for hypertension and cardiovascular disease screening. The exact mechanism underlying hypertension in people with prior exposure to nevirapine or thymidine analogues is unclear but may be related to metabolic changes. In the AGEhIV cohort, the association between stavudine exposure and hypertension was attenuated after adjustment for lipodystrophy [13], suggesting that lipodystrophy may have an underlying role. The mechanism underlying the elevated risk of hypertension associated with nevirapine use remains unclear and should be investigated further.

Our study analysis several limitations. First, there is potential residual confounding because we could not measure and adjust for other hypertension risk factors, such as family history, medication use, unhealthy diet, and physical inactivity. In addition, data on laboratory parameters were only available at baseline and could not be modelled as time-updated covariates. Second, we cannot rule out unmeasured biases, as the analysis is based on an observational cohort. Furthermore, blood pressure was only measured annually, and this may lead to, and this may lead to inaccurate estimation of blood pressure incident dates. However, most hypertension guidelines recommend at least one annual blood pressure measurement per year. Third, we did not determine the incidence of cardiovascular disease, an important complication of uncontrolled hypertension. However, the detection and management of cardiovascular disease remains a challenge for many HIV programs in SSA countries [8, 42]. Fourth, missing data for some variables could have resulted in bias, although the proportion of participants with missing data was consistently small across the variables, and data was completely missing at random. Finally, in our analysis, we could not determine the hypertension risk posed by contemporary ART regimens, such as INSTIs, as these agents were recently introduced in Uganda; therefore, such an analysis would be underpowered. Future studies should investigate the risk of hypertension posed by contemporary regimens in SSA cohorts since there are reports that agents are differentially associated with a greater risk of weight gain in black people [43].

Despite these limitations, our results may inform the improvement of hypertension and HIV-care programs in SSA. With the increasing survival of PLWH and the increasing burden of cardiovascular disease, screening and managing cardiovascular risk factors, such as hypertension, will become essential for preventing cardiovascular deaths, especially in SSA [6]. In the analysed cohort, the rates of other cardiovascular risk factors, such as diabetes and smoking, were low, signifying that hypertension may play an even more critical role as a cardiovascular risk factor in SSA. The low smoking rates are consistent with reports from other SSA HIV cohorts in which smoking rates are generally low in PLWH, especially in women [44]. In addition, data from longitudinal cohorts on the relationship between hypertension and antiretroviral exposure in PLWH with long ART exposure in SSA are lacking. Our cohort enrolled 1000 participants who had been on ART for at least 10 years at baseline and were subsequently followed up for almost eight years. Therefore, this analysis contributes to the evidence for screening for hypertension and its risk factors in PLWH with prolonged exposure to ART in SSA. Importantly, highlight the importance of blood pressure screening, supports lipid and glucose testing, and screening for renal insufficiency in heavily treated PLWH. Furthermore, this analysis has several strengths. This cohort had high retention, as 97% of the participants had at least two follow-up visits, thus minimising attrition bias. In addition, we used data from people with prolonged ART exposure, who reflect the future HIV population. Finally, the analysis was based on routinely collected clinical data and may represent real-life urban settings in Uganda.

In conclusion, the prevalence and incidence of hypertension in this cohort of heavily treated PLWH was higher than those previously reported in the same HIV clinic. In addition, hypertension was associated with traditional risk factors and exposure to older toxic antiretroviral agents. Collectively, our results highlight the need to monitor hypertension in PLWH with prolonged exposure to ART and the need for HIV programs in SSA to integrate hypertension screening into existing HIV treatment programs.

## Supporting information

S1 Fig Incidence rate of hypertension with increasing follow-up time. The rates of hypertension increased with increasing follow-up duration.
(TIF)
