# Supplementary figures and images for: Prevalence and incidence of hypertension in a heavily treatment-experienced cohort of people living with HIV in Uganda
Source: PLoS One. 2023 Feb 17;18(2):e0282001. doi: 10.1371/journal.pone.0282001 (PMC9937480; doi:10.1371/journal.pone.0282001)

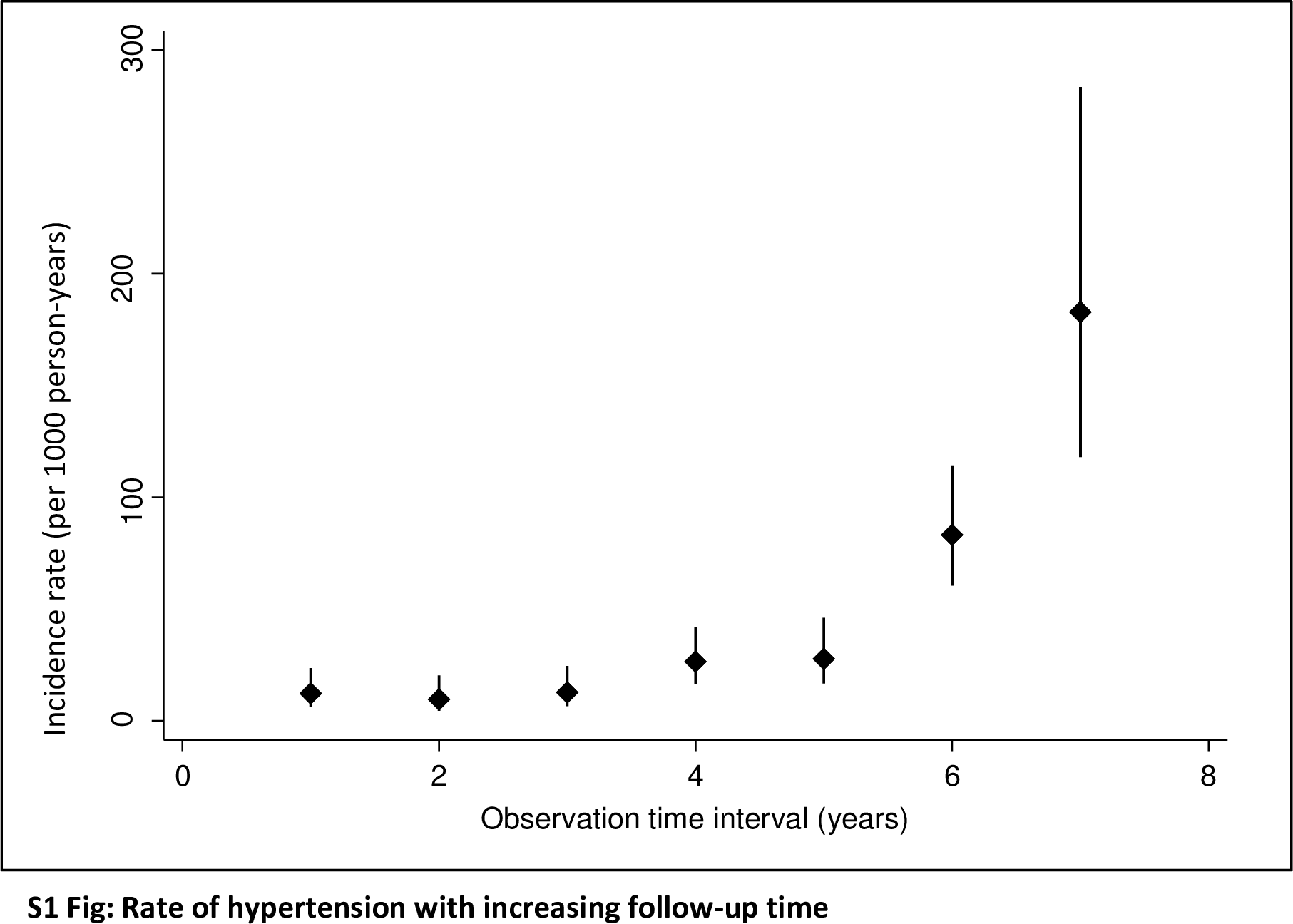

Supplement: S1 Fig — The rates of hypertension increased with increasing follow-up duration. (TIF) [file pone.0282001.s001.tif]
