# Supplementary material for: Prevalence and incidence of hypertension in a heavily treatment-experienced cohort of people living with HIV in Uganda
Source: PLoS One. 2023 Feb 17;18(2):e0282001. doi: 10.1371/journal.pone.0282001 (PMC9937480; doi:10.1371/journal.pone.0282001)
Supplement: Attachment — Submitted filename: Review.docx [file pone.0282001.s002.docx]

This study sought to determine the prevalence and incidence of hypertension and its relationship with exposure to specific antiretroviral therapy in a Ugandan cohort of PLWH with long durations of ART.

Minor

1. Define what is meant by “heavily treatment experience”.
2. Multivariable not multivariate.
3. The authors use rate ratios and risk ratios interchangeability yet these differ (Line 127).
4. The interpretation of odds ratios as percentages is not correct. (e.g., odds of prevalent hypertension increase by 16%).
5. Define contemporary ART regimens. Line 76.
6. What was considered as prior exposure to antiretroviral therapy in the staged model building approach line 115.
7. Provide references for definitions and analysis endpoints for example diabetes mellitus, hepatitis B, and chronic kidney disease.

**Major**

1. Clarify why those with more than two follow-up visits were excluded for the analysis of prevalence.
2. Was adherence to antiretroviral therapy considered in the analysis? How would it affect the estimates?
3. How was Cumulative exposure to first line and second-line ART modeled
4. For the logistic regression model how were the covariates chosen?
5. It seems counterintuitive to remove untreated viral drugs that were not independently associated with hypertension (line 117 -119) yet the objective of this study is to find the association between antiretroviral drugs and hypertension.
6. What was the rationale for choosing the non-ART confounders listed in line 121 - 122?
7. Stepwise variable selection modeling is problematic in many ways including: it yields confidence intervals for effects and predicted values that are falsely narrow (Altman and Andersen, 1989) and biased regression coefficients that need shrinkage (see Tibshirani, 1996).
8. Line 135. What was the evidence for the assumption of MAR?
9. Tamper the discussion and conclusions given that the blood pressure measurements were annual with a high possibility of measurement error and visit-visit variability.
10. Rate ratios on their own cannot be compared across populations. Instead use standardized rates for comparison of incidence rates the current study with those in other populations.
