# Supplementary material for: Prevalence and incidence of hypertension in a heavily treatment-experienced cohort of people living with HIV in Uganda
Source: PLoS One. 2023 Feb 17;18(2):e0282001. doi: 10.1371/journal.pone.0282001 (PMC9937480; doi:10.1371/journal.pone.0282001)
Supplement: Attachment — Submitted filename: Response to Reviewers.docx [file pone.0282001.s004.docx]

24^th^ January 2023

The Academic Editor, PLOS ONE

Dear Academic Editor,

**Re: RE:** **PONE-D-22-29381R1 Prevalence and incidence of hypertension in a heavily treatment-experienced cohort of people living with HIV in Uganda**

We want to thank the academic editor and reviewers for their consideration and opinions on the manuscript. We particularly thank the reviewer 1 for their useful comments and suggestions on improving the manuscript. We have addressed all comments and incorporated the suggestions into the revised manuscript where possible. Where we have differing opinion from the reviewer, we have indicated the reasons and provided references (last page of responses) in support of our position. Overall, we find the comments by the reviewers helpful, and we hope the revised manuscript meets your expectations for publication.

Below, we provide detailed point-by-point responses to the reviewers' comments. We have also tracked the changes in the revised manuscript.
Yours sincerely,

___________________________________

Dathan Byonanebye

On behalf of all authors

**REVIEWER # 1**

***General comments:***

1. ***General comment 1:*** Thank you very much for your detailed explanations of your choices and adding clarifications that unfortunately I missed when I read this through the first time. I still have some disagreements on a few comments that I will list below, at least partially because of poor phrasing in my initial review and the manuscript.

***Response:*** We thank the reviewer for carefully reviewing our responses and the revised manuscript. I particularly thank the reviewer for the scientific propositions and stimulating arguments. In our responses below, we address the specific issues raised by the reviewer.

2. ***Specific comment 1:*** As you have stated, you are exploring causality. That means you are relying on the sampling and the regression models to provide you with unbiased estimates of the causal effects. My "not systematically selected" comment was poorly phrased, but this is what I had in mind. Regression can do ok at providing unbiased estimates of causal effects in observational studies, but it's hard to know when regression does a good job without trying out other causal designs (e.g., propensity scores, disease risk scores, etc.). Thus, if your main goal is causality, it would seem to me to make sense to me to try one of these designs.

***Response:*** We understand the concern of the reviewer (see comment 3) is that we have used the term predictor, which in their view, suggests a causal relationship. We have made revisions to the manuscript and replaced "predictor" with factors associated". We understand the reviewer suggests that if we want to maintain the term "predictor," we should explore other statistical methods (e.g., propensity scores) as these, in their view, would better prove a causal relationship. Although propensity scores are increasingly used in statistical analyses, in our opinion, we do not agree that propensity scores are always superior to multivariate regressionIn a metanalysis of 43 observational studies Shah et al., demonstrated thatpropensity score methods give similar results to the conventional regression methods in observational studies [1]. In addition, Cepeda et al. demonstrated that the performance of propensity scores in large datasets with more than seven events per variable is similar to traditional multivariable regression methods [2] Therefore, we have maintained multivariate regression. However, we agree with the reviewer that the use of the term "predictors" may be strong in this case. To this end, we have revised the paper and dropped the use of "predictor" and used "factors associated".

3. ***Specific comment 2:*** In that vein, I have a hard time with this statement in your response: "…the aim of this analysis was to identify risk (or protective) factors that are causally related to hypertension…". I don't see how you can do that without having balanced groups at baseline. That's why I brought up age. I'm not disputing that age is a risk factor. I mentioned age as an example because, if the age distribution is different between the hypertension and non-hypertension groups at baseline, that could cause bias. Currently you are relying on the regression model to remove that bias, which it may or may not be able to do. In table 1, the median age is ~2.5 years higher in the hypertensive group. Unfortunately, I'm not knowledgeable enough in this area to know if that is that enough to make a difference. It might be nothing, but this is where having some sort of balancing procedure might be useful. I'm also not able to know whether the regression model is able to remove any confounding that may have (in this case, I suspect the regression model is based on the info in table 1 since there is good overlap).

***Response:*** We thank the reviewer for this comment. We have addressed some of the issues in this comment above. As we understand age, the reviewer's concerns are that there are baseline differences between participants who developed hypertension and those who did not. The reviewer points out (as an example) a 2.5-year difference in median age and suggests that some balancing procedure should be done. We do not agree with this suggestion. We aim to determine whether these differences are associated with a higher risk of hypertension. Balancing (e.g., matching participants by age) would remove the effect of age, which we would like to show. If matching is done for all known predictors of hypertension, then the analysis will likely not show any predictors, which is our analysis goal. As argued above we do not think that traditional multivariate regression methods are inferior to propensity scores, especially if the sample size is not small as in our case.

***Specific comment 3:*** Also, if you are not concerned with "prediction", I suggest not using the term "predictors" in the abstract, the title of Table 4, and in the discussion. Again, maybe it's just me, but this is where some of my dissonance arises.

***Response:*** We understand the genuine concern of the reviewer. We have revised the entire manuscript and dropped the use of "predictor" and used "factors associated". We hope the reviewer will find this change appropriate.

***Specific comment 4:*** Thank you for your detailed explanation of your variable selection. I agree with your general thesis that the variable selection literature is a mess, especially in epidemiology. If one goes back far enough, it is possible to find any reference to support any claim. Unfortunately, I still see what you are doing as a form of sequential variable selection based on this statement from the review: "Therefore, we fitted our model manually and variable selection was based on the significance of the variables and published data linking the variables to hypertension" and lines 143-145. You may not be using a specific algorithm and you may not be using "stepwise" where variables can exit and enter the model, but you are still using the p-value (which I presume is what you mean by "significance") to select variables in a sequential manner. From what I have seen from simulation studies, using p-values for selection with sequential model fitting does poorly at capturing the true effect unless the sample size is really large. That said, it's hard for me to assess incorporating the change-in-estimate and AIC into the selection procedure. I don't know conditional on the p-value based selection whether those reduce confounding and, if so, by how much. If we are working in an 'anything goes so long as you explain it' situation regarding variable selection, then I think what you have is permissible. But I am not really buying p-value based selection.

***Response:*** We thank the reviewer for reading our responses on this issue. The reviewer is concerned by the "sequential variable selection based" and is not sure whether "conditional on the p-value based selection whether those reduce confounding and, if so, by how much". We agree with the reviewer that variable selection solely based on p-value would be inappropriate even when done manually. However, ask the reviewer to note that our variable selection was not exclusively based on p-value alone. For example, we have clarified that we tested whether variables were confounders (cause >10% change in effect size) before dropping them from the model (lines 145-149). Specifically, we included confounders in the final model even if their p-values were insignificant. P-value-based procedures would have dropped these, and we agree that the estimates would not be robust. In each case, we also tested whether the model with the "non-significant" confounder fits the data better than the model without the confounder. In that case, we used change-in-estimate and AIC into the selection procedure. We admit this was not clear in the manuscript, and we have revised the manuscript to make this clearer in the methods. Regarding our statement in the previous responses, "Therefore, we fitted our model manually and variable selection was based on the significance of the variables and published data linking the variables to hypertension", we intended to show that confounders were fitted in the final model even when they would have been dropped based on p-value (because of non-significant p-value). To further be sure of the estimates, we have rerun a full model (with all variables stated apriori, regardless of bivariate p-value), and the results are broadly similar. Also, the reduced final model is better fitting than a full model(AIC 849.693 vs 855.7153). Therefore, we have maintained the reduced final model as this is better fitting to the analysed data.

**Reviewer 3: No comments**

**References**

1	Shah BR, Laupacis A, Hux JE, Austin PC. **Propensity score methods gave similar results to traditional regression modeling in observational studies: a systematic review**. *J Clin Epidemiol* 2005; **58**:550–559.

2	Cepeda MS, Boston R, Farrar JT, Strom BL. **Comparison of logistic regression versus propensity score when the number of events is low and there are multiple confounders**. *Am J Epidemiol* 2003; **158**:280–287.
